# Visualizing the lipid dynamics role in infrared neural stimulation using stimulated Raman scattering

**DOI:** 10.1016/j.bpj.2022.03.006

**Published:** 2022-03-08

**Authors:** Wilson R. Adams, Rekha Gautam, Andrea Locke, Laura E. Masson, Ana I. Borrachero-Conejo, Bryan R. Dollinger, Graham A. Throckmorton, Craig Duvall, E. Duco Jansen, Anita Mahadevan-Jansen

**Affiliations:** 1 Department of Biomedical Engineering, Vanderbilt University, Nashville, Tennessee; 2 Department of Neurosurgery, Vanderbilt University Medical Center, Nashville, Tennessee

## Abstract

Infrared neural stimulation (INS) uses pulsed infrared light to yield label-free neural stimulation with broad experimental and translational utility. Despite its robust demonstration, INS’s mechanistic and biophysical underpinnings have been the subject of debate for more than a decade. The role of lipid membrane thermodynamics appears to play an important role in how fast IR-mediated heating nonspecifically drives action potential generation. Direct observation of lipid membrane dynamics during INS remains to be shown in a live neural model system. We used hyperspectral stimulated Raman scattering microscopy to study biochemical signatures of high-speed vibrational dynamics underlying INS in a live neural cell culture model. The findings suggest that lipid bilayer structural changes occur during INS *in vitro* in NG108-15 neuroglioma cells. Lipid-specific signatures of cell stimulated Raman scattering spectra varied with stimulation energy and radiation exposure. The spectroscopic observations agree with high-speed ratiometric fluorescence imaging of a conventional lipophilic membrane structure reporter, 4-(2-(6-(dibutylamino)-2-naphthalenyl)ethenyl)-1-(3-sulfopropyl)pyridinium hydroxide. The findings support the hypothesis that INS causes changes in the lipid membrane of neural cells by changing the lipid membrane packing order. This work highlights the potential of hyperspectral stimulated Raman scattering as a method to safely study biophysical and biochemical dynamics in live cells.

## Significance

We used hyperspectral stimulated Raman scattering microscopy to study biochemical signatures of high-speed vibrational dynamics underlying infrared neural stimulation in a live neural cell culture model. Our findings highlight how leveraging lipid membrane dynamics can modulate cell function beyond just neurons. These observations lay the groundwork for developing faster and more precise neuromodulation approaches as we continue to understand brain function and treat its dysfunction.

## Introduction

Neuromodulation using directed energy, including optical, ultrasonic, and radio frequency, has attracted much attention because of its spatial precision, noninvasive implementation, and promising potential for clinical translation. Label-free optical neuromodulation with pulsed infrared (IR) light, or IR neural stimulation (INS), offers spatially and temporally precise means of contact-free activation of neural cells without the need for genetic modification or exogenous mediators. Like most label-free directed energy methods of neuromodulation, the biophysical mechanisms underlying INS have remained elusive for more than a decade (1) In contrast to the tools derived from molecular biology, such as optogenetics or photochemical uncaging, INS appears to act through an entirely different photothermally based mechanism (1,2). Lipid membrane dynamics appear to play an important role in how IR light depolarizes neurons photothermally (3) but remains to be directly experimentally observed in a live neural model system.

IR wavelengths generally used for INS are strongly absorbed by water (4,5). The rapid temperature rise from brief pulses of IR light depolarized HEK cells and synthetic charged lipid bilayer preparations through a transient increase in membrane capacitance (2). Biomolecular explanations for these observations are unclear. A biophysical explanation of this phenomenon has been described computationally by factoring in the thermal dependence of lipid bilayer geometry with a Gouy-Chapman-Stern-based electrodynamic model of charged lipid bilayers (3). Although the experimental data and computational model agree with each other, the role of lipid dynamics in neural models of INS remains to be directly validated. Lipid dynamics during INS have been probed through electrophysiology and fluorescent membrane structure reporters (2,6,7). However, these methods are inherently indirect to lipid molecular dynamics. There has not been any direct observation of lipid dynamics in live neural cells during INS. Understanding the role of lipid dynamics in the mechanisms of INS would provide valuable scientific insights and a basis for innovation toward the next generations of neuromodulation technology.

Conventional methods of directly measuring lipid bilayer geometry, such as x-ray diffraction and small-angle neutron scattering, are slow and not biologically compatible (8, 9, 10). Optical methods are well suited for high-resolution, biologically compatible experiments but generally lack the spatial resolution necessary to resolve lipid bilayer geometry (<3 nm thick) on millisecond timescales. Fluorescent functional lipid indicators, such as Laurdan or 4-(2-(6-(dibutylamino)-2-naphthalenyl)ethenyl)-1-(3-sulfopropyl)pyridinium hydroxide (di-4-ANNEPS) (11), are powerful tools for studying lipid membrane biophysics. However, these indicators offer latent readouts of lipid dynamics. Fluorescent reporters are inherently indirect, relying on the molecular interaction of reporter molecules with their molecular environment. Vibrational spectroscopic methods, such as Raman scattering and IR absorption, can be performed label free and offer a feature-rich molecular signature useful for studying lipid organization in live cells. Traditionally, vibrational spectroscopic methods have not been biologically compatible on sub-second timescales (12,13). Stimulated Raman scattering (SRS) microscopy combines label-free vibrational spectroscopic contrast with subcellular spatial resolution and sub-second temporal resolution, enabling time-resolved vibrational spectral measurements of live neural cells during INS (14,15). Others have shown that lipid molecular symmetry and ordered molecular interactions of water with lipid bilayers are observable with non-linear Raman microscopy (16,17). SRS imaging is fast enough to discern signatures of neuronal action potentials at millisecond timescales (16, 17, 18). With this in mind, we set out to employ a hyperspectral SRS (hsSRS) microscopy approach to identify vibrational signatures of lipid bilayer dynamics during INS in live neural cell cultures.

This study aims to identify the molecular dynamics of membrane lipids in live neural cells during INS with hsSRS microscopy. We demonstrate a time-resolved hsSRS methodology combined with focus precompensation to obtain SRS spectra of live NG108 cells. Spectral changes in NG108 cells are attributable to changes in the lipid packing order and solvent interactions. The observations agreed with gold-standard ratiometric fluorescence of a functional lipid packing order indicator, di-4-ANNEPS. We discuss how changes in cell vibrational spectral signatures during INS compare with what the current mechanistic hypothesis predicts. We also offer practical insights into performing high-resolution optical microscopy under dynamically varying optical imaging conditions during INS.

## Methods

### Cell culture and maintenance

Methods for neuronal hybridoma cell cultures were adapted from previous work (19,20). A spiking neuroma-glioblastoma hybridoma cell line, NG-108-15 (Sigma-Aldrich, St. Louis, MO), was thawed and maintained in culture for 1 week before imaging experiments. Cells were maintained in Dulbecco’s modified Eagle’s medium supplemented with 4.5 g/L of glucose, 20 mM L-glutamine, 15% (vol) fetal bovine serum, and 1% (vol) of penicillin/streptomycin antibiotics. Cells were incubated at 37°C in 5% of CO_2_ and 95% relative humidity. The growth medium was completely replaced every 48 h until cells approached confluency. When ∼80% confluent, cells were mechanically dissociated and propagated onto additional cell culture flasks until experimental use. All cells were imaged within 15 rounds of passage from thawed supplier stocks. Seventy-two hours prior to imaging, cells were passaged and plated onto poly-D-lysine-coated glass-bottom petri dishes (Mattek, Natick, MA) to allow cellular adherence. Twenty-four hours prior to imaging experiments, the cell culture medium was replaced with an identical Dulbecco’s modified Eagle’s medium formulation, except for the reduction of fetal bovine serum concentration (3% vol), to promote morphological differentiation into dendritic neuronal phenotypes. During imaging experiments, cells were maintained at room temperature and humidity in neurophysiologically balanced saline free of protein and glucose with the following composition: 140 mM NaCl, 4 mM KCl, 2 mM MgCl_2_, 2 mM CaCl_2_, 10 mM HEPES, and 5 mM glucose (pH 7.4) with NaOH and osmolarity adjusted to ∼318 mOsm with mannitol (21). Cells were imaged for 45 min before being discarded.

### Microscope system

**Figure 1:**
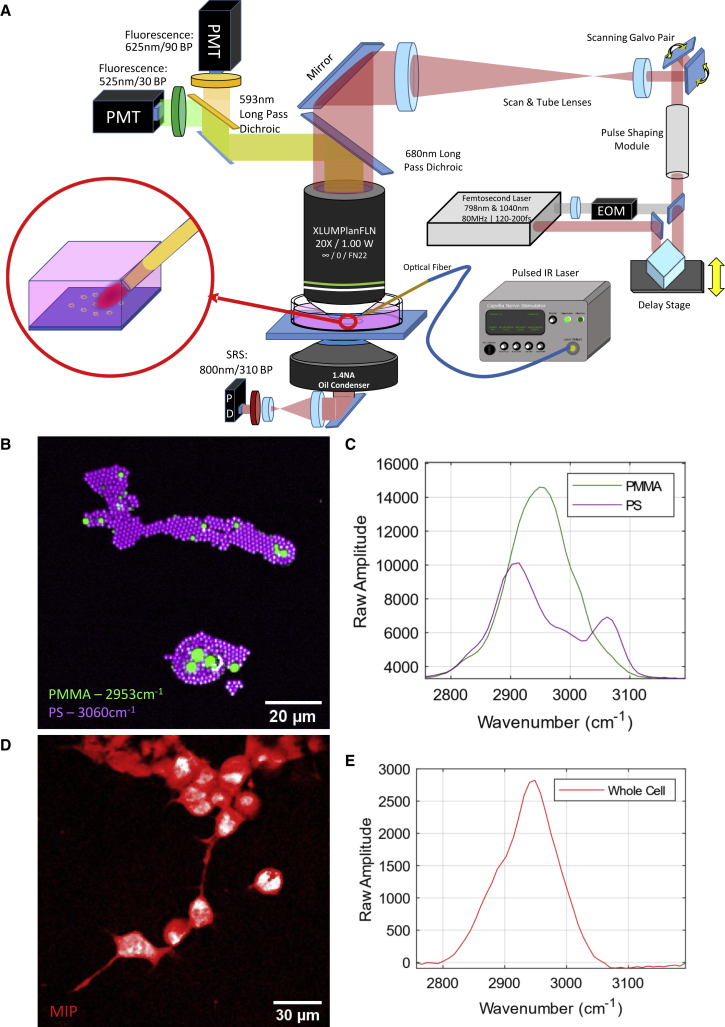
Experimental setup for SRS and fluorescence imaging of samples during IR exposure. (*A*) Imaging system schematic. (*B* and *C*) Standard poly(methyl methacrylate) and polystyrene (PMMA | PS) monolayer demonstrating spatial (B) and spectral (*C*) performance of the imaging system. (*D* and *E*) Maximum-intensity projection (MIP) of the hsSRS image stack of live NG108 cells (*D*) alongside their respective whole-cell SRS spectra (*E*), including cell somata and dendrites. To see figure color, go online.

The physical layout and capability of the custom-built multimodal imaging platform utilized in this study (Fig. 1 *A*) has been described previously (22). Briefly, a dual-output femtosecond near-IR laser source (Insight DS+, Spectra Physics, Santa Clara, CA, USA) was used to excite nonlinear contrast. Both output beams were spatially and temporally combined, with 20-MHz intensity modulation of the 1040-nm output and a variable linear optical path length on the 798-nm output for temporal collinearity and to facilitate hsSRS (23). The combined ultrafast laser outputs were subsequently chirped through 150-mm high-index SF11 glass rods (Newlight Photonics, Ontario, Canada) to enable spectral-focusing-based hsSRS microscopy (23,24). Chirping the two ultrafast laser pulses through high-index glass from ∼200 fs to about ∼2.5 ps allows tuning the relative time delay between the ultrafast laser pulses at the sample to variably evoke SRS resonances. The result is improved spectral resolution (∼30 cm^−1^) compared with using transform-limited 200-fs pulses (∼300 cm^−1^) without being limited by laser wavelength tuning speed. The result is a video rate nonlinear microscopy platform with 800-nm spatial resolution and approximately 30 cm^−1^ spectral resolution. After chirping, the beams were directed to a pair of scanning galvanometric mirrors. The face of the first scanning mirror was relayed to the back focal plane of a physiological imaging objective (Olympus XLUMPLN 20× 1.0 NA, water dipping) through a 4× magnifying 4-f imaging relay (SL50-2P and TL200-2P, Thorlabs, Newton, NJ).

Detection for SRS—specifically, stimulated Raman loss—was collected via transmission by a high-NA condenser lens (1.4 NA oil, Nikon Instruments, Melville, NY, USA.) directing light to a reverse-biased photodiode (APE, Berlin, Germany) behind an 850-nm centered, 310-nm bandwidth optical bandpass filter (Semrock, Brattleboro, VT) to isolate the 798-nm laser line. The detected signal was subsequently demodulated with a lock-in amplifier (APE) synced against the 20-MHz sinusoid signal driving the 1040-nm beam modulation. Any 20-MHz modulation transfer from the 1040-nm beam to the 798-nm beam was assumed to be attributed to stimulated Raman contrast. The temporal delay between the chirped 798-nm and 1040-nm laser pulses arriving at the sample was carefully tuned by varying the optical path length of the 798-nm laser beam with an optical delay stage (BB201, Thorlabs, Newton, NJ, USA). The relative delay between laser pulses over a span of 0.5 mm (or 1.6 ps) allowed scanning of SRS resonance contrast over approximately 300 cm^−1^ between 2800 and 3100 cm^−1^. This system also allows multiphoton fluorescence microscopy, which can be measured from epi-detected light reflected from a 680-nm long-pass dichroic mirror (Semrock) behind the objective lens in a non-descanned configuration. Bandpass filters for multiphoton fluorescence microscopy were selected to collect the green (525-nm center, 50-nm passband) and red (625-nm center, 90-nm passband) emission profiles of the lipophilic dye di-4-ANNEPS. Images were acquired in a bidirectional point-scanning configuration. High-speed hsSRS imaging experiments were acquired with a 96 × 64 pixel (px) sampling profile with varying pixel sampling densities between 1 and 4 μm/px. Imaging with 2–5 μs pixel dwell time and bidirectional scanning amounts to an effective imaging frame rate approaching 150 Hz. Ultrafast laser average powers at the sample plane were measured to be about 10 mW for the 798-nm laser and 25 mW for the 1040-nm laser, corresponding to 199 watts (W)/cm^2^ and 497 W/cm^2^ irradiances at each pixel, respectively. Assuming a constant 5 μs pixel dwell time, radiation exposure per pixel amounted to 1.00 mJ/cm^2^ and 2.49 mJ/cm^2^. The substantial increase in power necessary for imaging in this configuration is due to the decrease in peak power within each ultrafast laser pulse during the chirping stage. Live-cell viability was verified with all relevant imaging conditions by cellular uptake of propidium iodide (PI) and is described under “Live-cell hsSRS imaging.”

### hsSRS spectral focusing calibration

A monolayer preparation of mixed polymer beads was used to calibrate the optical delay between pump and probe laser pulses as a function of SRS resonance. A mixed sample of poly(methyl methacrylate) (PMMA; 1–10 μm in diameter) and polystyrene (PS; 2 μm in diameter) microspheres (PolySciences, Warrington, PA, USA) was diluted to a concentration of 0.002% (w/v) each in a solution of methanol (Fisherbrand, St. Louis, MO, USA). After mixing, 10 μL of diluted microbead solution was spread onto a #1.5 glass coverslip and left to evaporate for 25 min at room temperature. When dry, samples were mounted dry onto a standard microscope slide and used for spectral calibration of the hsSRS system by spectral focusing.

Sequential images (*n* = 50) of mounted polymer bead monolayers were acquired to calibrate the vibrational spectral dimension of the hyperspectral imaging space. Between each acquired image, the optical path length delay of the 798-nm laser line was stepped by 10 μm between each image over a total of 500 μm or 1.6 ps of total optical path length delay. The peak SRS signal for the 2950-cm^−1^ resonance of PMMA was centered in the spectral scanning range to ensure sufficient spectral sampling. Manual segmentation of PMMA and PS beads from spectral stacks was performed and averaged across each spectral frame to provide high-fidelity spectra for both polymers. The known vibrational peaks of PS (2910 and 3060 cm^−1^) and PMMA (2950 cm^−1^) were used as spectral fiducials (Fig. 1, *B* and *C*) to linearly interpolate a relationship between the optical path delay of the chirped 798-nm laser pulse and the excited vibrational resonant mode. Calibrations were performed at the beginning of each day’s experiments to ensure spectral accuracy. The spectral resolution was observed to be approximately 30 cm^−1^.

### INS

Neural stimulation was performed by placing a bare 400-μm-diameter core low-OH optical fiber (Ocean Optics, Orlando, FL, USA) in close proximity to samples (∼450 μm) at a 30° approach angle into the sample plane of the microscope’s field of view (Fig. S1). The optical fiber used for stimulation is connected to a pulsed laser diode centered at 1875 nm (Capella Nerve Stimulator, Aculight; Lockheed-Martin, Bothel, WA, USA). During imaging experiments, samples were exposed to a pulse train of 188 pulses distributed evenly over 1500 ms. Pulses were 400 μs in duration and delivered at a repetition rate of 125 Hz. Radiation exposure of samples was varied by adjusting the peak current delivered to the laser diode, holding all dosing and geometric configurations constant. Radiation exposure calculations for stimulation were approximated based on power measurements performed externally in air and employing Beer’s law under the assumption of an absorption-dominated photon distribution, as described in Figs. S1 and S2. IR exposure levels for INS were selected based on their ability to elicit dynamic calcium responses (>2% increase, dF/F) in NG108 cells loaded with a calcium dye (Fluo-4-AM at 1 μM; Thermo Fisher, St. Louis, MO). Radiation exposure for no stimulation, sub-threshold, and threshold levels of stimulation used 0, 5.02, and 10.63 J/cm^2^, respectively.

### Phospholipid multilamellar vesicle preparation

Multi-lamellar vesicles were used to obtain lipid-derived SRS spectra free of protein and nucleic acids signal in a biomimetic context. Multi-lamellar vesicles were prepared according to protocols provided by the supplier (Avanti Polar Lipids, Alabaster, AL, USA). Phosphatidylcholine (PC) derived from porcine brain tissue arrived dissolved in chloroform at a concentration of 2.5 mg/mL. The chloroform was evaporated from the lipid mixture with a stream of dry nitrogen overnight and mechanically resolubilized in phosphate-buffered saline (PBS) solution at a concentration of 1 mg/mL. Vesicle mixtures were stored at 4°C and imaged within 3 days of preparation. Imaging was performed at room temperature. Size distribution of the lipid vesicle preparation was verified via dynamic light scattering to contain 1- and 5-μm-diameter vesicles (Malvern Panalytical, Malvern, UK). Multi-lamellar vesicles (MLVs) were identified as multilayered spherical structures with SRS contrast tuned to 2910 cm^−1^ (Fig. S4 *A*).

### Live-cell hsSRS imaging

Live-cell imaging experiments of endogenous vibrational contrast with hsSRS were conducted with adherent cell preparations imaged in a physiologically balanced saline solution. Following placement of the fiber and calibration of the spectral axis against the known vibrational peaks of PS and PMMA beads, baseline hyperspectral image stacks were acquired for live-cell samples. All images were acquired in a point-scanning approach with a 5-μs pixel dwell time and a spatial sampling density of ∼500 nm/px. To improve the signal-to-noise ratio of higher fidelity images, square fields of view between 320 and 512 px in size were acquired, and 6 to 10 images were averaged together for each spectral position. For hyperspectral image stack acquisitions, 50 images were acquired at evenly spaced intervals (10 μm) over 500 μm of optical path length delay, corresponding to a spectral range spanning approximately 2800–3100 cm^−1^. The resultant spectral image stack was taken as the ground-truth cellular spectrum to compare high-speed imaging spectra of the cells during INS in subsequent experiments.

**Figure 2:**
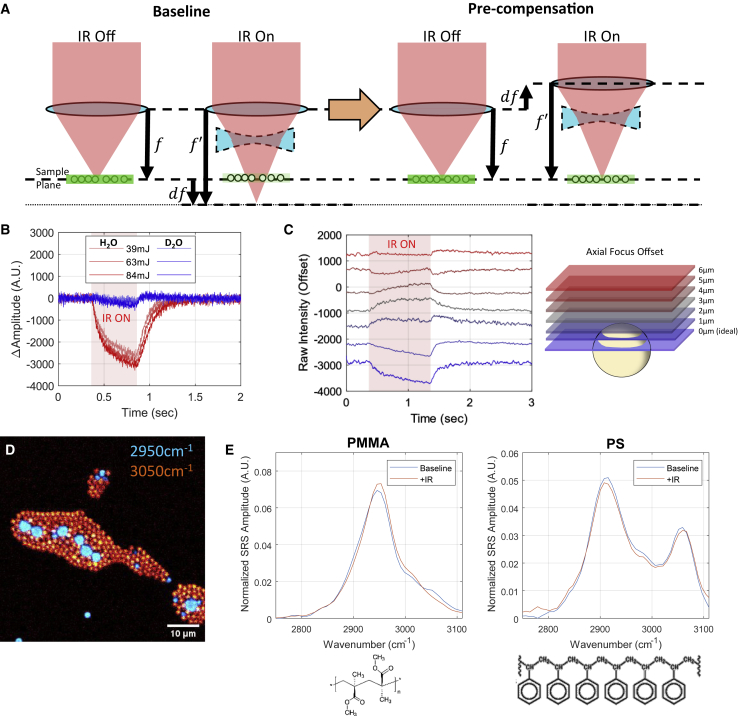
Explanation of the defocusing phenomenon and the proposed experimental approach to circumvent it. (*A*) By adjusting the microscope focal plane to accommodate focal shifts induced by pulsed-IR neurostimulation within the microscope’s field of view, it is possible to recover some lost nonlinear signal because of defocusing. (*B*) The thermal gradient and subsequent defocusing artifact generated by INS in the microscope’s field of view is due to water absorption of INS light. Replacing H_2_O immersion with D_2_O immersion for imaging demonstrates that absorption of IR light is the driving force behind defocusing and signal loss. (*C*) Pre-compensating for INS-induced defocus by adjusting the focal plane position relative to our sample allows nonlinear signal during INS. (*D*–*F*) Extrapolating this experimental approach across the wave number ROIs allows reconstruction of vibrational spectral dynamics during fast biophysical thermal events such as INS. (*D*) Composite SRS image of PMMA and PS beads at 2950 and 3050 cm^−1^, respectively. (*E* and *F*) Baseline and IR-stimulated spectra for (*E*) PMMA and (*F*) PS, reconstructed using the focus precompensation approach, with the respective chemical structures for reference. To see figure color, go online.

**Figure 3:**
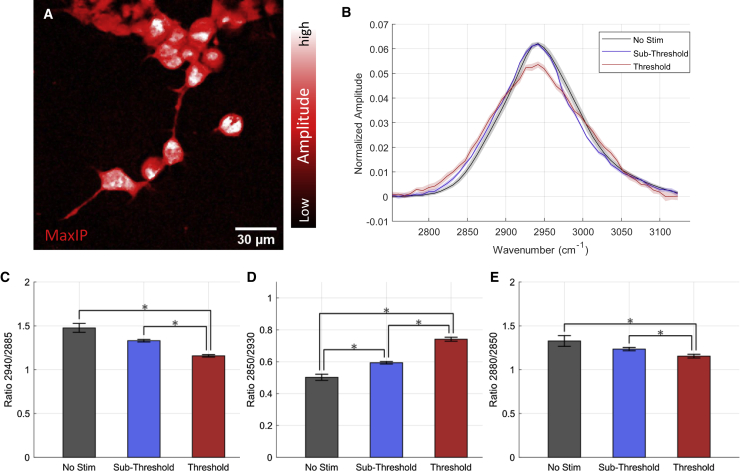
Vibrational spectroscopic imaging of NG108 cells during INS. (*A*) MIP of an NG108 spectral image stack from 2800–3150 cm^−1^ (*n* = 50 images). (*B*) Average SRS spectra obtained from NG108 cells during INS at and above activation threshold radiation exposure (*n* = 10–24 cells per group). (*C–E*) Peak ratio comparisons indicating (*C*) asCH_2_/asCH_3_ as a measure of *trans-gauche* isomerization of lipid tail groups, (*D*) symCH_2_/symCH_3_ as a measure of increased polar headgroup association with water because of membrane packing order decrease, and (*E*) asCH_2_/symCH_2_ as an indicator of decreasing acyl-chain packing order. ^∗^p < 0.05. To see figure color, go online.

For high-speed imaging during INS on NG108 cells as well as control samples of MLVs and bovine serum albumin (BSA) solution, a 5-μs pixel dwell time was employed to obtain imaging fields 96 × 64 px in size with a sampling density between 1.5 and 4 μm/px, enabling frame rates of 33.4 Hz. For each of the 50 spectral position, cells were imaged continuously for 5 s, during which a train of stimulating IR pulses was delivered at the first second of the imaging time frame. Image acquisition and IR stimulation were coordinated through a customized transistor-transistor logig (TTL) triggering protocol with an external signal digitizer (Digidata 1550B; Molecular Devices, Sunnyvale, CA). The ultrafast excitation laser was observed to defocus at the sample plane due to the thermal gradient induced by the stimulating IR laser (Fig. 2 *A*). This was observable in each imaging time series as an exponential decrease, and subsequent return to baseline (Fig. 2, *B* and *C*), of the nonlinear signal during imaging. The shift in focal length as a function of laser power was calibrated using microbead (PMMA and PS) preparations and accounted for prior to each IR stimulation trial on cells. The defocusing phenomenon allowed precise temporal synchronization of time series across each spectral channel. After repeating and temporally aligning simultaneous imaging and stimulation time courses on live cells for each SRS spectral position (*n* = 50), the temporal evolution of live-cell endogenous vibrational spectra could be observed as a function of irradiation time and deposited energy. For spectral evaluation, the final 10 sampling time points the IR exposure window were averaged to help reduce high frequency spectral noise from influencing conclusions. Spectra from stimulation experiments were pooled from 24 cells across 10 distinct experiments of IR exposure. Regions from cell somata and dendrites were included in the segmentation process when present. Each cell spectrum was normalized with respect to its integrated spectral intensity, and standard deviations of the spectra across all cells under each stimulation condition were calculated. The “no stimulation” conditions were obtained from the initial SRS signal from cells prior to each round of IR exposure and pooled from all stimulation conditions being compared. The shape of SRS spectra acquired at high frame rates (Fig. 3 *B*) were not found to noticeably differ from higher-fidelity spectra (Fig. 1 *D*).

To verify cell viability during IR exposure, NG108 cells were subjected to the hsSRS and stimulation protocol described above while monitoring cell damage via positive fluorescence staining of cell nuclei with PI. Imaging protocols were identical to those described previously while supplementing the cell imaging medium with 1 μM PI (Thermo Fisher). Cell morphology was monitored throughout the experiment by comparing high-fidelity images (<1 μm/px sampling density) of the cells before and after imaging at their peak SRS resonance contrast at 2930 cm^−1^.

### di-4-ANNEPS ratiometric fluorescence imaging

Imaging protocols were adapted from work published previously (25). Briefly, a loading solution of di-4-ANNEPS was prepared by diluting an aliquot of 4 mM stock solution in dimethyl sulfoxide in neurophysiological saline to a final loading concentration of 2 μM. NG108 cells were incubated in the dark at 37°C, 5% CO_2_, and 95% relative humidity for 25 min before being rinsed and maintained in fresh neurophysiological saline solution free of dye for fluorescence imaging. To image di-4-ANNEPS fluorescence, two photomultiplier tubes were configured for non-descanned epifluorescence detection. Fluorescence emission was split by a 593-nm long-pass dichroic mirror and subsequently filtered with a 525-nm/25 or 625-nm/45 optical bandpass filter before reaching the photomultiplier tube detectors (Semrock). Ultrafast laser excitation for multiphoton fluorescence was tuned to 960 nm to optimally excite di-4-ANNEPS. For high-speed imaging, images were acquired as 96 × 64 px images between 0.5 and 4.0 μm/px sampling densities with 5-μs pixel dwell times to yield 33.4 Hz frame rates. Excitation laser intensity for imaging was maintained below 10 mW at the sample plane. The SF11 glass rods used to chirp the laser pulses for hsSRS imaging were removed for ratiometric fluorescence imaging, resulting in ultrafast laser pulse width approaching 200 fs at approximately 80 MHz.

During a 5-s imaging period, stimulating IR light was delivered to di-4-ANNEPS-stained NG108 cells via a 400-μm core multimode optical fiber immediately adjacent to the microscope’s field of view. Varying levels of radiation exposure were delivered to cells (0–44 J/cm^2^), and the resulting fluorescence intensity changes were compared across stimulation conditions. Calculations for conventional polarization as well as a modified version of general polarization (Fig. S6) were derived to compare conventional assessments of lipid packing with that observed with hsSRS.

### Data processing, analysis, and visualization

#### hsSRS imaging data

Raw data acquired from the imaging experiments were collated and sorted into multidimensional stacks of 16-bit tagged image file format (TIFF) stacks separated by time and wave number using a customize processing pipeline in Fiji leveraging the Bioformats plugin (26,27). Average intensity projections of multidimensional (spectral and temporal) image stacks in time and spectral space were used to generate a mask to segment cells geometries. A general region of interest identified from the resultant masks was applied to the raw multidimensional image stack to extract spectral and temporal data from features of interest (e.g., beads and cells). To segment individual cells, a 2-px Gaussian blur was applied to the average intensity projection of the multidimensional image stack, and contrast local histogram equalization was performed to reduce cell signal intensity variations between cells. Post hoc flat field correction of imaging field heterogeneity of images was implemented by scaling pixel intensities relative to the average intensity projection Gaussian blurred with a kernel equal to 0.25–0.5× the largest dimension of a particular image. Prominent peak locations in the image were identified. The filtered average intensity projection was subsequently segmented via Otsu segmentation. The resulting mask and previously identified peak locations were fed into a seeded watershed segmentation algorithm that reliably separates and segments individual cells as their own regions of interest (ROIs) with minimal cell-to-cell overlap (28). Edge maps of cells were acquired by subtracting the watershed-segmented mask from itself following an erosion operation, which reliably identifies borders in a cell-specific manner. The resultant ROIs are applied to the raw stacks to extract the mean amplitude, the standard deviation of signal or amplitude measurements, and their respective centroid locations in image space for each spatial and temporal point. This process is automated as a macro procedure in Fiji and is freely available upon request. Images provided in the manuscript are derived from single frames at specific wave numbers of interest or maximum intensity projections of spectral image stacks. For visualization purposes in publication, intensity scaling for all images was adjusted linearly.

All hsSRS spectra are smoothed with a three-point sliding Gaussian window and normalized with respect to their integrated spectral area. Because the study intends to compare the relative spectral shapes of each sample, integrated spectral normalization was chosen to facilitate this interpretation. Error associated with each plot is presented as the standard deviation of all averaged spectra obtained for a given experimental trial. Each bead was taken as one sample, and different trials were taken as independent observations for statistical analysis purposes. For peak ratio comparisons, vibrational resonance intensities were calculated utilizing a cubic spline interpolation of the measured spectral data and its respective standard deviation. Comparisons of peak ratios were assessed using a Student’s two-sided *t*-test, where errors associated with ratiometric comparisons were calculated based on the propagation of error of the interpolated standard deviations (statistical significance was denoted by ^∗^p < 0.05 and ^∗∗^p < 0.01). All quantitative work was performed in MATLAB (Mathworks, Natick, MA) using native functions. All bar graphs were created using the Superbar package.

### Ratiometric fluorescence analysis of di-4-ANNEPS data

Processing of ratiometric fluorescence data is derived in part from previous work (29). Raw image stacks of green (lipid membrane gel phase - ordered) and red (lipid membrane liquid phase - disordered) spectral emission channels are acquired simultaneously at a 33.4-Hz frame rate. Conventional general polarization (GP_conv_) was calculated using the following equation:(29)

$$GP_{conv}\left(t\right)=\frac{\left(O\left(t\right)-D\left(t\right)\right)}{\left(O\left(t\right)+D\left(t\right)\right)}.$$

The raw image intensity differences between the green (ordered, O(t)) and red (disordered, D(t)) imaging channels were divided by the sum of both channels for each time point in the image stack for each experiment. Decreases in GP_conv_ value generally suggest decreases in membrane packing order. Average GP values as a function of time were calculated, and each cell’s GP value was taken as an average GP of all pixels contained in each cell’s ROI. Cell segmentation similar to that segmented for SRS images utilizing a seeded watershed method was performed. However, because di-4-ANNEPS preferentially labels the extracellular membrane, a Huang threshold mask of raw disordered spectral fluorescence intensity images was obtained to determine cell boundaries, and a binary fill operation was employed to identify areas in the image that contained cells. The lack of lipid-stained fluorescence in cell nuclei was used to identify the center points of cells. The raw disordered fluorescence channel image was smoothed with a 2-px Gaussian filter, and local minima in the images were used to approximately localize cell center points. These cell center points, as well as the cell position mask and a distance map calculated from the cell position mask, were fed into a seeded watershed algorithm in Fiji to yield segmentation maps of individual cells in a given experiment (26,28). The ROIs derived from the segmentation were subsequently applied to each imaging experiment, where time series of raw fluorescence channels were obtained per cell and the resultant data were exported for processing and analysis in MATLAB (Mathworks). Statistical comparison of GP values across stimulation conditions was performed using a two-sided Student’s *t*-test, and the magnitudes and standard error of means across the GP values were calculated across all individual cells in a particular experimental condition (statistical significance denoted as ^∗^p < 0.05).

**Figure 4:**
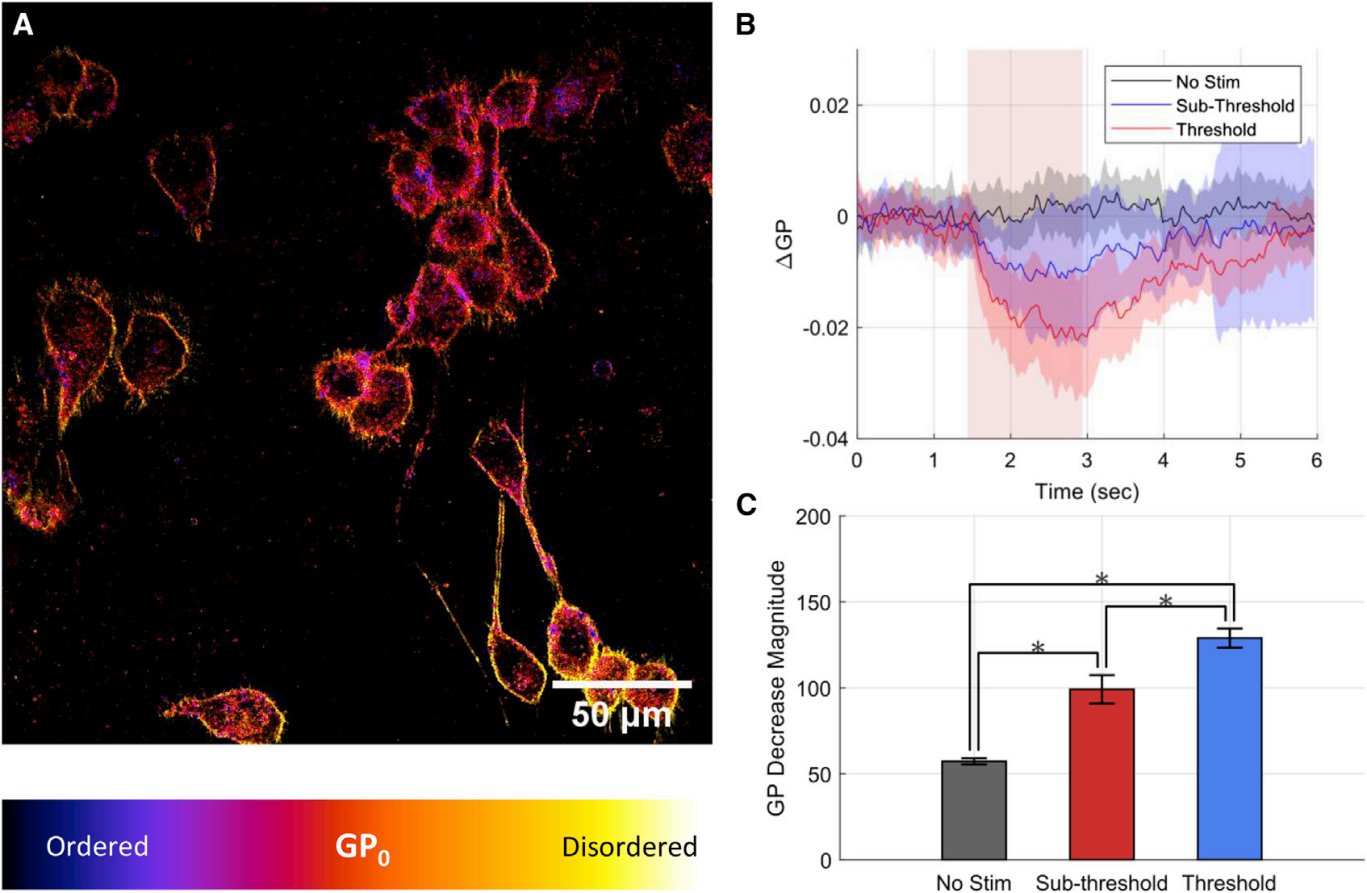
Relative changes in general polarization (GP) measurements in NG108 cells, measuring dual-band fluorescence of di-4-ANNEPS to verify changes in membrane order during INS. (*A*) Fluorescence intensity images overlaid with calculated initial GP values of NG108 cell cultures loaded with di-4-ANNEPS. (*B*) Relative changes in adapted GP metrics in NG108 cells during various doses of IR stimulation. Decreases in relative GP are indicative of decreases in the relative extracellular lipid membrane packing order, which agree with hsSRS observations. Error traces represent standard deviation across all cell responses (*n* = 50–109 cells). (*C*) Magnitude of GP decreases across sub-threshold (5.02 J/cm^2^) and threshold (10.63 J/cm^2^) levels of radiation exposure. Error bars represent SEM across all cells within each condition. ^∗^p < 0.05.

For image visualization, adapted from previous work (29), 8-bit-depth raw fluorescence intensity images from the disordered fluorescence channel were multiplied by each color channel of a red-green-blue format image representing the calculated GP images with the desired false-colored lookup table of preference. The resulting images yield an image where pixel brightness represents intensity and color represents calculated GP_conv_, which is used purely for visualization purposes. All rescaling of intensities in images are linear and performed for clarity of cellular morphologies and biophysical properties in print (Fig. 4 *A*).

Because of large variations in total fluorescence measured in any given experiment as a result of thermal lensing during IR stimulation, the conventional method of calculating GP was found to be unreliable. Because we expect a decrease in overall fluorescence as a result of the decrease in effective collection efficiency during thermal lensing-induced defocusing, the magnitude of changes in the denominator of the GP_conv_ equation is much larger than that of the changes in the numerator of the equation. We developed an intensity-invariant version of GP_conv_ to better reflect these dynamics mathematically over short experimental periods of time undergoing substantial changes in photon collection:

$$GP_{mod}\left(t\right)=\frac{\left[O_{0}-D_{0}\right]+\left[O_{off}\left(t\right)-D_{off}\left(t\right)\right]}{\left[O_{0}+D_{0}\right]}$$

where O_0_ represents initial ordered fluorescence levels, D_0_ represents initial disordered fluorescence levels

$$O_{off}\left(t\right)=\left[O\left(t\right)-O_{0}\right]\;$$

$$D_{off}\left(t\right)=\left[D\left(t\right)-D_{0}\right]\;$$

O_off_(t) represents the net change in ordered fluorescence relative to O_0_ as a function of time, and D_off_(t) represents the net change in disordered fluorescence as a function of time. O(t) and D(t) are the raw ordered and disordered fluorescences as a function of time, respectively (Fig. S6 *B*). The alternative metric of modified GP (GP_mod_) emphasizes the raw difference in measured fluorescence intensity between the ordered and disordered fluorescence imaging channels without dividing by the sum of both image channels over time. Assuming the defocusing artifact between both channels results in an equal amount of defocusing and signal loss from each fluorescence channel, any change in the relative difference between the fluorescence signals as a function of time is indicative of functional changes in lipid bilayer packing (Fig. S6). For the purposes of this study, we are interested in determining the direction of GP changes, positive or negative, rather than its magnitude. This consideration makes GP_mod_ a convenient and applicable tool for our experimental approach.

## Results

### Thermal lensing during IR stimulation

Following confirmation of our instrument’s ability to obtain hsSRS image stacks from live NG108 cells (Fig. 1, *D* and *E*), initial experiments with IR stimulation during nonlinear microscopy (i.e., any coherent Raman modality, multiphoton fluorescence, or higher harmonic generation) resulted in a substantial loss in measured signal during IR exposure (Fig. 2, *B* and *C*) (22). This was apparent in short periods of heating from a millisecond pulse of IR light (unpublished data) and pulse trains of multiple microsecond pulses of light. The shape of the disappearance and reappearance of the nonlinear signal appears to follow the shape of the expected heating and cooling dynamics that are typically observed during IR-mediated heating (30), suggesting that a temperature-related phenomenon may be responsible for the loss in signal. Considering that the goal of this work is to image the high-speed chemical dynamics in live cells during IR exposure, the loss of signal during this critical period posed a challenge. To better understand the role of this signal loss in immersion medium temperature, a vegetable oil sample was imaged with SRS (2885 cm^−1^) through warmed immersion medium at a range of physiologically relevant temperatures. The temperature of the immersion medium was monitored by a thermocouple placed adjacent to the microscope’s field of view at the coverglass-immersion medium interface (Fig. S3). Warmed deionized water (approximately 50°C) was added between the objective and sample, with the edge of the vegetable oil sample placed in focus. Images were acquired continuously as the immersion medium slowly cooled to room temperature (22°C). Contrary to the signal decrease observed during rapid IR heating (Fig. 2, *B* and *C*), this experiment showed that changes in immersion medium temperature correlated positively with the temperature and SRS signal of vegetable oil. These data suggested that changes in immersion medium temperature alone were not sufficient to explain the decrease in nonlinear optical signal during IR heating.

The refractive index of the objective immersion medium, water (H_2_O), is correlated negatively with temperature (31). This concept suggests that the spatial thermal gradients generated by the IR absorption from IR stimulation would defocus the ultrafast laser driving nonlinear contrast and, thus, reduce the observed nonlinear optical signal. To test this hypothesis, the immersion medium for the objective lens was replaced with heavy water (D_2_O), which has a fivefold lower absorption coefficient at 1875 nm than deionized water, with nearly identical refractive indices (2). If the thermal gradient causes the decrease in nonlinear signal observed in the sample, then reducing the immersion medium’s IR absorption properties should reduce the magnitude of the nonlinear signal decrease during stimulation. The results shown in Fig. 2 *B* validate this hypothesis (Fig. 2 *B*), suggesting that the thermal gradient from IR stimulation defocused the ultrafast laser source, resulting in a decrease in nonlinear signal (Fig. 2 *A*).

`Because water’s index of refraction is correlated negatively with temperature, the thermal gradient generated during IR stimulation in front of the stimulation fiber and within the microscope’s field of view behaves like a negative lens during imaging. Imaging out-of-focus samples during IR stimulation would bring samples into focus (Fig. 2 *A*). This hypothesis was valid for nonlinear imaging and IR transillumination imaging. By moving the microscope’s focal plane above the sample by a few micrometers before IR exposure, the samples (polymer microbeads in this case) would come into focus (Fig. 2 *C*). This precompensation of defocus was applied repeatedly across numerous spectral channels to generate a time-resolved hsSRS profile of samples during IR stimulation, similar to approaches employed previously with hsSRS and electrophysiology (18,32). This approach was verified by measuring several control samples: PS/PMMA microbead monolayer mixtures, 10% (w/v) BSA solution in PBS, and large MLVs of neurologically derived PC and phosphatidylethanolamine in physiologically balanced neural saline solution before conducting experiments using live cellular samples.

### Verifying pre-compensation for thermal defocusing during hsSRS

Figure 2*D* shows a representative image of mixed microbead monolayers, highlighting PMMA in cyan using the band at 2950 cm^−1^(terminal methyl C-H resonance) and PS in orange using the band at 3050 cm^−1^ (aromatic C-H stretch resonance). The mixed-bead sample was exposed to ∼12 J/cm^2^ IR stimulation, and the resultant spectra for both bead types are shown in Fig. 2, *E* and *F*. Relevant spectral band assignments for polymer microbead samples are summarized in Table 1 (33, 34, 35). IR-exposed PMMA beads exhibit several distinct spectral changes upon heating: decreases in the 2880- and 2910-cm^−1^ resonances of skeletal C-H stretching as well as relative increases in resonances at 3000 cm^−1^ and decreases at 3050 cm^−1^. Shifts in PS hsSRS spectra during IR exposure show relative increased vibrational activity around 2850 cm^−1^, implying the possibility of relaxed steric hindrance of skeletal sp^3^ (alkane) C-H symmetric stretching modes. Broadening in the 3050 cm^−1^ peak is attributable to aromatic sp^2^ (alkene) C-H stretching and suggests reduced steric hindrance around aromatic side chains. These observations show that utilizing a time-resolved approach to obtaining hsSRS spectra of samples heated by pulsed IR light is feasible in highly Raman-active idealized chemical samples.

**Table 1:** Raman spectral band assignments in the CH-stretch region for control and cellular samples

Wave number	Chemical	Assignment
**Polymer microbeads**		

2847	PMMA	C-H stretching of O-CH_3_
2885	PMMA	C-H stretching of α-CH_3_
2910	PMMA	symmetric C-H of -CH_2_, C-H stretching of O-CH_3_
2950	PMMA	symmetric C-H of α-CH_3_, symmetric C-H of O-CH_3_, asymmetric C-H of -CH_2_
3000	PMMA	asymmetric C-H of O-CH_3_, asymmetric C-H of α-CH_3_
3050	PMMA	asymmetric C-H of O-CH_3_
2850	PS	symmetric C-H of CH_2_
2915	PS	asymmetric C-H of CH_2_
3050	PS	HC—H stretching of aromatic ring

**Biological lipids**		

2850	lipids	symmetric C-H stretch of aliphatic -CH_2_
2880 or 2885	lipids	asymmetric C-H of aliphatic -CH_2_, Fermi resonance between the symmetric C-H stretching mode and the overtone of the C-H bending vibrations
2970 or 2960	lipids	asymmetric C-H stretch of -CH3
3015 or 3023	lipids	alkyl HC—H stretches

**Biological proteins**		

		symmetric C-H stretch of -CH_3_
		asymmetric C-H stretch of -CH_2_
2940 or 2930	proteins	2930 cm^−1^ corresponds to the overtone of the CH_2_ scissoring (δ(CH2)) enhanced by Fermi resonance with the v_s_-(CH_2_) mode
3000–3060	proteins	sp^2^ C-H stretch of aromatic/vinyl amino acid residues (HC—H)

The dominant Raman scatterers in the 2800- to 3100-cm^−1^ spectral region primarily include lipids and proteins, with some marginal nucleic acid contributions (36,37). Spatially and spectrally, nucleic acids are easy to separate in cellular images (38). However, because proteins and lipids in cells do not appear as spatially distinct at the resolution of our microscope, their distinct spectral information must be used to draw conclusions about their molecular dynamics. Understanding how proteins and lipids are separately affected by IR stimulation provides insight into the spectral shifts that can be attributed to each biomolecule during live-cell imaging. hsSRS imaging with IR stimulation was performed on separate aqueous preparations of biomimetic MLVs (PC, neurologically derived, porcine sourced, Avanti Polar Lipids) and BSA (10% (w/v)) solutions.

An emulsion of MLVs was imaged with hsSRS and focus precompensation during radiation exposure equivalent to threshold levels (10.63 J/cm^2^) of IR exposures in live cells. These vesicles serve as a coarse chemical representation of cells to provide an isolated lipid preparation free of protein or carbohydrate contributions to vibrational spectra. IR-exposed MLV spectra (Fig. S4, *A* and *B*) show distinct shifts in lipid molecule resonance relevant to the lipid molecular packing order. Relevant spectral band assignments for biological lipid samples are summarized in Table 1. The 2850-cm^−1^ symmetric aliphatic C-H stretch resonance is markedly decreased, along with its Fermi resonance at 2880 cm^−1^. sp2 C-H stretching resonances associated with unsaturated aliphatic chain motifs at 3010 cm^−1^ are substantially decreased. Crucially, ratiometric comparison of 2880 and 2850 cm^−1^ shows reduced rotational restriction in alkane chains or decreased aliphatic tail packing order within the hydrophobic region of the membrane (Fig. S4 *C*). This is supported by the observed decrease in the ratio from 2940 to 2830 cm^−1^, which relates to increases in the solvent interaction with lipids (Fig. S4 *C*). These observations suggest that thermodynamic changes in lipid vibrational signatures during IR stimulation are discernable with hsSRS.

The edge of a 10% (w/v) BSA solution meniscus was imaged with hsSRS using radiation exposure equivalent to threshold levels of IR exposure in live cells to characterize protein vibrational signature changes during IR-induced heating (Fig. S4 *D*). Changes in protein spectra during IR exposure appear to be negligible (Fig. S4 *E*). The contribution of protein vibrational spectra in ratiometric comparisons that reveal significant changes in MLV samples appear to contribute negligibly to IR exposure changes in the BSA sample (Fig. S4 *F*). The amino acid constituents of BSA, a water-soluble protein, may not be directly representative of a transmembrane protein one would observe as a component of the extracellular membrane or intracellular organelles. However, the data support previous work showing that the shape of protein spectra in the CH-stretch region of the Raman spectrum does not appreciably change with temperature (39,40).

### hsSRS of neural cell models during INS

hsSRS imaging during IR stimulation was conducted in an *in vitro* neural cell model, a neuroma-glioblastoma hybridoma cell line (NG-108-15, Sigma-Aldrich). The NG-108 cell line is a practically robust and experimentally resilient neuronal cell model for hsSRS imaging. These cells are an accepted electrodynamic model of *in vitro* neurons and have been used successfully to study electrodynamics evoked by IR stimulation (19,20,41). Fig. 3 *A* shows a maximum intensity projection of a hsSRS spectral image stack to highlight the morphology of NG-108 cells. Successful stimulation with pulsed IR light was verified in separate experiments (unpublished data) of NG108 cells loaded with a calcium-sensitive dye, Fluo-4-AM, at 1 μM in balanced saline for 45 min. Two-photon fluorescence and SRS images centered at 2880 cm^−1^, an asymmetric sp3 CH_2_ resonance dominantly from lipids, were acquired simultaneously during IR stimulation of NG108 cells at a range of IR doses until noticeable increases in calcium-dependent fluorescence responses were evoked (>2% increase in dF/F). Levels of IR radiation evoking consistent intracellular calcium responses across the microscope’s field of view were referred to as threshold levels of exposure. Cells were imaged with hsSRS during IR stimulation with threshold and subthreshold (about half of threshold levels) doses of IR light.

The resultant area-normalized hsSRS spectra of NG108 cells under baseline (unstimulated), subthreshold, and threshold stimulation conditions are shown in Fig. 3 *B*. Relevant spectral band assignments for biological samples in the CH-stretch region are summarized in Table 1. Shoulders appearing at 2850 cm^−1^ during stimulation indicate relatively increased vibrational resonant activity from symmetric aliphatic C-H stretching in lipid tail chains. Decreases in the relative intensity ratio between 2940 and 2885 cm^−1^ (Fig. 3 *C*) suggest a decrease in packing order within the hydrocarbon tails of the lipid molecules because of *trans*-*gauche* isomerization of sp^3^ hydrocarbon chains. Interestingly, the 2850 cm^−1^ shoulder appears to increase in spectral intensity relative to the associated Fermi resonance at 2880 cm^−1^, possibly suggesting a reduction of intermolecular steric hindrance between aliphatic lipid tails or more rotational freedom of hydrocarbon chains. These observations were quantified by calculating the intensity ratio between 2850 and 2940 cm^−1^ (Fig. 3 *D*) as well as 2880 and 2850 cm^−1^ (Fig. 3 *E*). These metrics offer a quantification of lipid tail chain packing order, which has been hypothesized previously to decrease during IR stimulation (3). Statistically significant differences (^∗^p < 0.05) in these ratios suggest decreased hydrocarbon tail chain packing in cellular lipid membranes. For each comparison, the ratios calculated for subthreshold exposure fall between unstimulated and stimulated conditions. In particular, the shoulder around 3030 cm^−1^ – an sp2 CH (methylene) resonance assignable to CH bonds at points of unsaturation in lipid hydrocarbon tails, appears at the threshold stimulation but is reduced in the subthreshold and “no stimulation” cases (Fig. 3 *B*).

As described above, the hsSRS spectral acquisition requires cells to be exposed to 50 different rounds of IR stimulation, possibly damaging the cells and yielding biologically irrelevant observations. Although no morphological changes were observed in the stimulation experiments, cell viability was verified after repeated IR exposure. Exposed NG108 cells were imaged with multiphoton fluorescence to track the uptake of a cell damage indicator, PI, simultaneous with SRS tuned to the 2940 cm^−1^ CH_3_ resonance. Cells were imaged through 50 rounds of stimulation using parameters similar to those used during a live-cell hsSRS imaging experiment (Fig. S5 *A*). Some cell swelling was observed morphologically, but no uptake of PI was observed (Fig. S5 *B*), suggesting that the repetitive nature of hsSRS acquisition did not have any effect on acute cell viability.

### Ratiometric fluorescence imaging of a functional lipid dye during INS to verify changes in the lipid bilayer packing order

Ratiometric fluorescence of di-4-ANNEPS emission, a probe of the membrane packing order, was employed to verify cellular lipid dynamics as observed in vibrational spectra (25). Di-4-ANNEPS rotoisomerization is known to be dependent on the fatty acid tail packing order in lipid membranes. During IR stimulation, if the lipid tail packing order is decreased, then a similar decrease in the GP metric should follow. In place of the conventional approach for calculating GP, intensity-invariant adaptation of GP was utilized to circumvent the defocusing effect during IR stimulation (detailed in Methods and Fig. S6). Fig. 4 *A* depicts an intensity image of di-4-ANNEPS-loaded NG-108 cells overlaid with the color denoting GP calculation at each pixel. Fig. 4, *B* and *C*, shows the mean single-cell GP time traces and their standard deviations for each dosing condition. The intensity-invariant GP of di-4-ANNEPS (Fig. S6; Methods) shows a substantial decrease in GP as a function of IR stimulation dosage (Fig. 4 *C*). A decrease in GP suggests a decrease in the lipid chain packing order during IR stimulation, supporting the hsSRS observations.

## Discussion

Our current understanding of label-free directed energy neuromodulation continues to raise questions about their mechanistic bases. An improved understanding of INS mechanisms provides a fundamental framework for development of future innovative neuromodulation technologies. Here we provide an approach that uses hsSRS microscopy to gain insights into the role of lipid dynamics in live neural cells during INS. Most traditional methods to observe lipid-specific dynamics (e.g., isolated lipid preparations, electrophysiology, x-ray diffraction, and neutron scattering) in cells in real time suffer from a lack of specificity or biological compatibility. Methods that utilize fluorescent tags (e.g., fluorescence correlation spectroscopy and fluorescence recovery after photobleaching) provide insights into the dynamics of lipids in live cells but are inherently indirect. The goal of this work was to directly observe the biophysical dynamics of INS with a vibrational spectroscopic approach in live neural cells. Using the intrinsic Raman contrast of lipids, spectroscopic insight would help clarify the mechanistic role of lipid dynamics in INS. Our demonstration of characterizing and precompensating for dynamic defocus during INS with hsSRS is a novel approach in biomedical microscopy that applies to studying the molecular biophysics of live-cell models more generally.

Photothermal events are notoriously difficult to address with biological microscopy because of the relationship between temperature and refractive index in water. Although bulk changes in sample temperature can affect optical aberrations in microscopes, spatial thermal gradients that vary on the order of the microscope’s field of view can significantly affect the refraction of light into the sample (Fig. 2 *B*). Accounting for defocusing actively on millisecond timescales may be possible with dynamic adaptive optics approaches but is far from trivial to implement. Instead, our approach to adjust for IR-induced defocusing of the fluorescence excitation empirically (Fig. 2, *A* and *C*), although coarse compared with adaptive optics, enables us to gather useful insight into the biophysical phenomena associated with INS (Fig. 3). The reliable timing of stimulation can be leveraged to employ a time-resolved spectroscopy approach to hsSRS imaging at high frame rates. We demonstrate that high-speed vibrational dynamics can be resolved safely in live-cell preparations to yield biologically meaningful observations. When studying INS using high-NA microscopy, where IR-induced deflections in focal length can equal or exceed the depth of focus of a particular imaging objective, we urge others to interpret their results cautiously. Thermal defocusing can have a disproportionate effect on single-channel intensity measurements and must be accounted for carefully (Fig. S6). In cases where intensity noticeably changes during exposure, we encourage others to employ ratiometric or multi-spectral approaches to allow defocusing artifacts to be corrected. With fluorescence microscopy, where quantum yield, fluorescence intensity, and spectral profiles are well known to be sensitive to heating and defocusing (42, 43, 44), having simultaneous or time-resolved multi-spectral reference bases will allow such artifacts to be addressed in post-processing.

There are several spectral changes in the CH-stretch region of the Raman spectrum (2800–3100 cm^−1^) that one might expect to see if the current INS mechanistic model is valid. *Trans*-*gauche* isomerization, or rotoisomerization, of sp3 hydrocarbon chains, primarily associated with lipid hydrophobic tail groups in Raman imaging, can give rise to steric effects that drive lipid membrane deformations (39,40,45,46). Specifically, the lipid packing order, or the ability of lipid molecules to stack neatly alongside each other within the membrane leaflets, has been hypothesized to decrease with elevated temperature during INS. Rotoisomerization in membrane lipids geometrically shortens acyl tail groups, resulting in membrane thinning. Although quantifying the absolute deformation of lipid membrane thickness with SRS would require additional calibration experiments, hsSRS can quantify relative indicators of molecular interactions. An increased quantity of gauche rotamers within the hydrophobic region of the membrane leads to geometric acyl tail shortening and sterically drives lipid molecules apart from each other. The result is a decrease in the membrane packing order. In the CH-stretch region of the Raman spectrum, relative changes in symmetric (2850 cm^−1^) and asymmetric (2880 cm^−1^) aliphatic C-H stretching indicate shifts in the molecular packing order because of changes in the rotational freedom of hydrocarbon chains in lipid tails. The Raman signal at these resonances is primarily attributed to biological lipids (Fig. S4) (36). A decrease in the ratio of 2880 and 2850 cm^−1^ during INS (Fig. 3 *E*) is indicative of a “loose” packing order between lipid molecules or an increase in *trans-gauche* isomerization (47,48). Rotoisomerization of lipid tails is well known to decrease membrane thickness and increase the area of each lipid molecule’s solvent interactions (49, 50, 51). Changes in the ratio between 2940 and 2885 cm^−1^ offer insights into water interaction with lipid molecules, which should increase with temperature. The data show a decrease in the ratio between 2940 and 2885 cm^−1^ (Fig. 3 *C*), which is in line with the idea that lipid molecules expand within the membrane leaflets to leave room for more potential solvent interactions (e.g., hydrogen bonding) with elevated temperature. The IR dose dependence of this observation suggests that the relative degree of isomerization correlates with levels of IR exposure that would evoke neural activity *in vitro*. A progressive increase in isomerization with IR exposure supports INS’s existing mechanistic model, where changes in physical bilayer geometry accompany transient temperature changes.

The shoulder appearing around 2990 and 3030 cm^−1^ during INS in cells (Fig. 3 *B*) arises from relative increases in vinyl C-H resonances, which correspond to points of unsaturation in lipid-tail acyl chains. Relative increases in vinyl C-H signal can arise from the reduced steric hindrance of sp2 C-H stretching as well as compositional or membrane potential-related changes when the lipid bilayer undergoes thermal changes. Curiously, the 3030 cm^−1^ shoulder appearing in threshold stimulated cell spectra was reduced in sub-threshold stimulation levels. This resonance at 3030 cm^−1^ may provide a key marker for neural biophysics during INS.

The vinyl portion (2980–3100 cm^−1^) of the C-H stretch region contains SRS signal contributions from proteins, particularly from amino acid residues such as tyrosine, phenylalanine, and tryptophan. These amino acids play a key structural role in stabilizing hydrophobic domains of transmembrane proteins in the cell membrane. Our control experiments (Fig. S4) as well as evidence from others (39,40,52) reinforce that thermal changes in proteins are not significant contributors in the CH stretch region of the Raman spectrum. Therefore, we conclude that the protein signal contributes minimally to the photothermally mediated changes in cell SRS spectra during INS. Others have assigned relative decreases in the 2930-cm^−1^ signal to changes in cellular membrane potential, enabling visualization of neuronal action potentials with SRS microscopy (18,32). These spectral changes were attributed to the decrease in positively charged proteins accumulating electrostatically at the extracellular membrane surface when a cell is at its resting membrane potential. A reduction in membrane potential was suspected to reduce membrane-associated proteins in solution at the cell membrane surface. Our results show a considerable reduction in relative 2930- to 2940-cm^−1^ signal during INS (Fig. 3 *B*); thus, the electrostatic association of soluble proteins with cell surfaces may play some role in our results. Several experimental details suggest that membrane potential changes from electrostatic protein association would not contribute to our spectra. Defocusing artifacts make it challenging to reach conclusions about absolute molecular concentrations during INS (Fig. 2, *A*–*C*). Practically, our approach to ROI selection, non-balanced detection, and imaging medium formulation confounds any comparability of our results with these previous studies. However, Lee et al. did employ a similar time-resolved approach for acquiring SRS spectra as a function of membrane potential, demonstrating the utility of such an approach for certain types of experiments beyond photothermal phenomena (18).

The physical changes in the lipid bilayer during rapid heating with IR light are thought, at least in part, to give rise to the cell capacitance increase that drives cellular depolarization during INS (2,3). Our results (Fig. 3) support the idea that the lipid bilayer undergoes chemical changes during INS. These changes are observable with hsSRS and correlate with the level of the delivered stimulus. Although these findings are promising, they do not definitively support the hypothesis that bilayer deformation is directly causal of the stimulatory effect of INS. Although beyond the scope of this work, questions remain about how transmembrane ion channels may be independently sensitive to lipid membrane geometry and thermodynamics. Lipid thermodynamics are known to affect the conformational and functional properties of transmembrane ion channels (53, 54, 55, 56). It is unclear whether the capacitive effect or the actual physical change in the lipid bilayers themselves gives rise to stimulatory phenomena; decoupling the chemophysical and thermal electrodynamic changes in biologically relevant preparations would provide insights into this. Preparations of lipid vesicles or cells expressing voltage-gated ion channels loaded with a UV photo-switchable lipid analog (e.g., containing an azobenzene moiety in the tail group) may be a helpful set of experiments. The photo-switching property of such synthetic lipids would allow optical control of the membrane packing order with substantially reduced photothermal effects.

The current hypothesis for how INS occurs is that rapid heating causes a capacitive inward current that can depolarize neurons and lead to action potential generation (2). This capacitive current is thought to arise from biophysical changes within the extracellular membrane, *trans*-*gauche* isomerization of lipid acyl tail chains, that change the physical dimensions of the extracellular membrane because of temperature elevations (3). This deformation is accompanied by a movement of membrane-associated charge that, when hot and fast enough, can generate an inward current that depolarizes cells. The model of this phenomenon relies on steady-state chemical measurements of synthetic lipid bilayer geometry (57,58). The changes in bilayer geometry are used to inform a computational electrodynamic model that is compared against previous experimental work (2,41). Although the model of chemophysical and electrodynamic phenomena convincingly reproduces experimental data, capacitance changes and cellular electrodynamics are ultimately influenced by more than lipid dynamics alone *in vitro* and *in vivo*. Our work here provides direct evidence showing that lipid membranes dynamically change during INS in neural cells. The causality of this phenomenon remains to be proven. Nevertheless, the insights provided by our work shows how lipid membrane dynamics can be leveraged selectively to modulate cellular physiology.

Our SRS spectral observations are supported by an additional gold-standard means of measuring lipid dynamics in real time: the ratiometric fluorescence of a lipophilic dye, di-4-ANNEPS (Figs. 4 and S6). The negative changes in GP during INS affirm the decrease in membrane packing order observed with hsSRS. The magnitude of the changes in GP scaled with the level of stimulus delivered (Fig. 4, *B* and *C*). The data further suggest that hsSRS can be leveraged as a complementary tool to study lipid biophysics alongside traditional fluorescence approaches. Others have applied hsSRS to observe lipid biophysics in synthetic preparations (16,17) or to study lipid metabolism at the biomolecular level (59,60). Stimulated Raman microscopy has not been applied to study biological thermodynamics at sub-second timescales. Our work explores a temporal regime of live-cell biophysics that few have ventured into with SRS. This study provides a practical extension of hsSRS development while shedding light on a question pertinent to the field of optical neuromodulation.

Although implementation of hsSRS here can resolve high-speed spectral dynamics well below a second, it does take several minutes to build observations of events on a spectral basis. In situations where repeated perturbation of cells is not practical, the same approach can be implemented with a drastically reduced number of spectral channels. Alternatively, multi-spectral approaches leveraging the simultaneous acquisition of multiple resonances would be advantageous. At least two spectral channels need to be acquired simultaneously to circumvent the defocusing artifacts described here; single-shot and hysteresis-prone perturbations are not readily applicable with the approach demonstrated here. The rapid development rates of SRS in bioimaging show promise for pushing SRS-based methods to their limits. Our work shows that hsSRS can be applied to a range of lipid biophysics experiments to complement more conventional fluorescence-based approaches.

In contrast to fluorescence-based approaches that rely on the indirect readout from reporter molecules interacting with lipids in the cell membrane, vibrational contrast like that of hsSRS enables direct inference to be made specific to lipids at the intra- and intermolecular levels. As coherent Raman imaging continues to improve with better lasers, detectors, and signal processing strategies, we can expect to see extensions of hsSRS to address many other areas of lipid biophysics and beyond. Currently, signal to noise limits the real-time performance of hsSRS in the fingerprint region of the Raman spectrum (400–1700 cm^−1^). In future studies, we aim to study the fingerprint resonances that provide information about other biomolecules, such as DNA, RNA, and carbohydrates. Such approaches could help study macromolecular phase separation phenomena, chromatin dynamics, or glycogen metabolism directly without exogenous labeling. Coherent Raman imaging can be readily performed simultaneously with other nonlinear microscopy modalities (22). Multiplexing modalities might enable studies of how lipid membrane biophysics can influence biological dynamics with conventionally accepted molecular reporters. With this in mind, hsSRS has promising potential for a diverse range of bioimaging applications.

Alternative approaches utilizing deuterated lipid preparations to shift lipid-specific resonances into the “silent window” of the Raman spectrum (1700–2700 cm^−1^) may offer additional insight into the role of vinyl C-D resonances in the biophysics of INS (12,61, 62, 63). However, applications of deuterated lipids may not be easily replicable in live cells because they can interfere with the hydrogen bonding dynamics crucial for cell membrane integrity. Currently, implementing rapid hsSRS is technically hampered by the signal-to-noise performance in the fingerprint window of the Raman spectrum (400–1700 cm^−1^). Utilizing other features of the Raman spectrum that are more directly attributed to lipid-tail chain rotoisomerization (e.g., the skeletal vibrational C-C modes between 1030 and 1150 cm^−1^ as well as C=C stretching modes around 1650 cm^−1^) might provide more direct mechanistic insights to INS when possible (47). Some promising newer spectroscopic and computational denoising methods that circumvent these noise issues are gaining popularity but still require careful validation for high-speed imaging of cellular dynamics (64, 65, 66, 67). Ongoing work continues to improve the technical capabilities of SRS so that real-time imaging of fingerprint spectral features in live cells may be possible. Coherent anti-Stokes Raman scattering (CARS), a contrast modality similar to SRS – has achieved considerably fast imaging throughput at a high spectral resolution over the span of the CARS spectrum (5ms/px dwell times over >3000 cm^−1^ bandwidth) (64,68) Although this approach was too slow for spatially resolving cellular dynamics in real time for our study, broadband CARS approaches may be suitable for numerous other biological applications with different instrument performance needs.

Although the data presented here offer support for involvement of lipid dynamics in INS, it needs to be noted that focus precompensation and hsSRS do not readily show the absolute magnitude of deformation in the cell membrane during INS. With a molecular dynamic model of INS biophysics, simple bilayer geometry simulations may enable some degree of calibration to correlate observed hsSRS spectra with lipid bilayer physical properties. Without explicit approximations of lipid bilayer physical or electrical properties, it becomes difficult to judge or estimate the cell capacitance changes postulated to depolarize cells from SRS data alone. Integrating voltage imaging or electrophysiology alongside our existing hsSRS experimental preparation may help identify a relationship between lipid dynamics and capacitance. Imaging systems with frame rates exceeding 1 kHz can provide a window into these dynamics; however, we were unable to reach such high frame rates with our system without damaging cells. Our results provide supportive evidence of the role of lipids in INS; however, the data do not show a causal relationship between lipid dynamics and INS. Our imaging approach does not differentiate between extracellular membranes and intracellular organelle membranes. Transmembrane protein sensitivity to INS phenomena is still not clear, although it is known that different molecular pathways can be actuated depending on cell phenotype (20,21,69, 70, 71, 72, 73). Despite these caveats, the provided data demonstrate that lipid bilayer dynamics are changing during INS and that these changes track with the magnitude of the stimulus. These results provide validation of the current mechanism’s key assumptions in a live neural cell model. The understanding of this concept serves as a crucial basis for understanding label-free neuromodulation more broadly. The general experimental framework presented here is readily applicable to other methods of directed energy neuromodulation as well as study of other dynamic processes.

The mechanistic basis of directed energy label-free neuromodulation has long been a question lacking complete answers (74,75). Having a better understanding of how directed energy in the optical domain can be used to modulate brain function opens the door for innovation in neuromodulation to improve spatial targeting, temporal accuracy, and long-term utility, optically or otherwise. Extending this understanding to developing new neuromodulation methods, neural prostheses, and therapeutic interventions is a promising outlook for directed energy approaches. Whether the mechanistic bases for methods of directed energy neuromodulation, such as IR, ultrasonic, or radio frequency-based approaches, are shared remains to be demonstrated. Our approach may serve as a valuable benchmark for answering such questions as technology in neuromodulation and hsSRS imaging continues to develop.

## Conclusion

We used hsSRS to experimentally demonstrate the mechanistic involvement of lipid dynamics in INS in live neural cells. Our results provide direct supportive evidence of lipid bilayer structural changes related to thermally induced *trans-gauche* isomerization of lipid-tail hydrocarbon chains during INS. These experimental observations are in line with the currently proposed mechanistic model of INS. Our results reinforce the idea that the photothermal basis of INS may be driving a general, nonspecific effect in live cells that evokes a multitude of physiological responses. The experimental framework also highlights the utility of hsSRS microscopy in addressing questions with high temporal resolution requirements and will continue to provide information about live-cell biophysics beyond neuromodulation.

## Data availability

Any raw or processed data, processing, and analysis code is available upon request from the corresponding authors.

## Author contributions

A.M.-J., E.D.J., and W.R.A. conceived the idea for the manuscript. A.M.-J. and E.D.J. secured funding support for the published work. A.M.-J., E.D.J., G.A.T., R.G., and W.R.A. designed the experiments. A.L. assisted with identifying, preparing, and imaging the control samples for the study and interpreting the results. A.I.B.-C. assisted with preparing cell cultures, formulating experimental approaches, and data analysis. R.G., A.L., and G.A.T. contributed to data processing and analysis. B.R.D., C.D., A.L., and W.R.A. prepared the multi-lamellar vesicles. W.R.A. assisted with all sample preparations; performed all imaging experiments, image processing, data analyses, and data visualization; and wrote the manuscript. All authors contributed to editing the manuscript.

## Supporting material

Document S1. Figures S1–S7

Document S2. Article plus supporting material
