# Supplementary material for: Visualizing the lipid dynamics role in infrared neural stimulation using stimulated Raman scattering
Source: Biophys J. 2022 Mar 8;121(8):1525–40. doi: 10.1016/j.bpj.2022.03.006 (PMC9072573; doi:10.1016/j.bpj.2022.03.006)
Supplement: Document S1. Figures S1–S7 [file mmc1.pdf]

Biophysical Journal, Volume *121*

Supplemental information

Visualizing the lipid dynamics role in infrared neural stimulation using stimulated Raman scattering

Wilson R. Adams, Rekha Gautam, Andrea Locke, Laura E. Masson, Ana I. Borrachero-Conejo, Bryan R. Dollinger, Graham A. Throckmorton, Craig Duvall, E. Duco Jansen, and Anita Mahadevan-Jansen

Title
Visualizing Lipid Dynamics Role in Infrared Neural Stimulation using Stimulated Raman Scattering

Authors
Wilson R Adams[1], Rekha Gautam[1], Andrea Locke[1], Laura E Masson[1], Ana I. Borrachero-Conejo[1], Bryan Dollinger[1], Graham A. Throckmorton[1], Craig Duvall[1], E Duco Jansen[1,2], Anita Mahadevan-Jansen[1,2,*]
* Corresponding Author: anita.mahadevan-jansen@vanderbilt.edu

Affiliations
[1] Dept. of Biomedical Engineering, Vanderbilt University, Nashville, TN, USA
[2] Dept. of Neurosurgery, Vanderbilt University Medical Center, Nashville, TN, USA

Supporting Material

Figure S1: A**)** Illumination geometry and B) calculation of approximate fiber distance for estimating radiant exposure – where d_{fiber} is the optical fiber diameter, r_{fiber} is the optical fiber radius, θ_A is the fiber approach angle, d_{cs+} is the fiber edge's distance off of the surface of the cover slip, and l is the normal distance from the optical fiber face to the cover slip plane.

Figure S2: Optical dosage calculations at the cell imaging plane based on an absorption-dominated photon distribution in homogenous medium, assuming negligible scattering and non-angled fiber approach to the sample

Figure S3: Temperature dependence of 2930 cm^{-1} CARS and SRS signal. A) experimental imaging and temperature measurement setup. B) Raw intensity measurements of vegetable oil meniscus as a function of temperature.

Figure S4: Validation of IR stimulated hsSRS images on isolated control sample preparations of major biological Raman scatterers. (A) SRS image of multi-lamellar vesicles at 2930 cm^{-1} resonance. (B) SRS spectra of baseline and IR-stimulated MLVs. (C, F) Ratiometric comparison of MLVs and BSA SRS spectra, respectively, of resonances indicative of lipid membrane biophysical dynamics.

Figure S5: NG108 Cell Viability following hsSRS and repeated INS – (A) Representative average intensity projection images of NG108 cells with SRS (left, magenta) and 2P fluorescence (green, right, identical intensity image scaling) of a cell viability indicator, propidium iodide (PI). Slight differences in cell morphology appear after 50 rounds of INS (bottom) compared to 1 round of INS (top). No substantial update of PI was observable. Scale bars are all 40 μm in width. (B) Intensity level comparison of PI fluorescence in cells exposed to different amounts of threshold INS events. No significant differences observed between non-stimulated and stimulated conditions. Significantly lower fluorescence compared to positive control of dead cells across all conditions. Asterisk indicates $p < 0.05$ based on a 2-sided student's t-test comparisons of cell intensity means and standard deviations across all measured cells ($n = 38$).

Figure S6: An intensity-invariant metric of general polarization for di-4-ANNEPS imaging of cells during IR stimulation, where signal loss from thermal lensing significantly impacts perceived signal interpretation. A) di-4-ANNEPS loaded NG108 cells. B) Baseline-offset mean detected intensities of mean disordered (black line) and ordered (red line) of all cells in a given experiment, plotted alongside the difference of detected intensities (Ordered – Disordered) C) Calculated conventional general polarization timeseries during IR stimulation alongside adapted general polarization calculation. D) Conventional and adapted GP metric calculations alongside each other. Eliminating the time dependance of the denominator term circumvents the defocusing artifact's impact on the GP calculation.

Figure S7: Sub-band analysis of cell SRS spectra based on multi-gaussian curve fitting – A) An example cell spectrum (red) with its corresponding fit (black, dashed) and sub-band constituents. B) Vibrational resonance center frequencies included in the fitting process – derived from Czamara et al. J

Raman Spectroscopy 2014. C) Intensity comparison of each sub-band to cell SRS spectra under different stimulation conditions. Error bars represent standard deviation of individual cell spectra.

Supplemental Figures

$$d_{fiber} = 440\mu m \qquad r_f = 220\mu m$$

$$\theta_A = 30° \qquad sin\,\theta_A = \frac{1}{2} \qquad \cos\theta_A = \frac{\sqrt{3}}{2}$$

$$d_{cs} = 10\mu m$$

$$l = \frac{(r_f \cos\theta_A) + d_{cs}}{\sin\theta_A} = 400\mu m$$

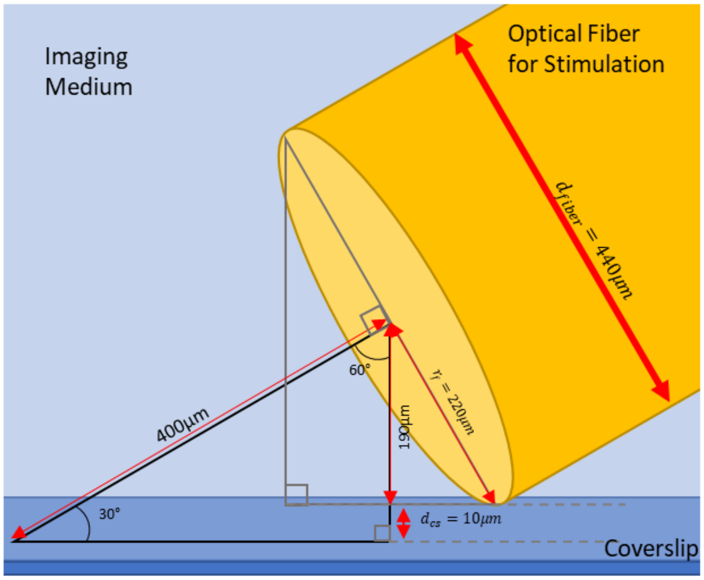

Figure S1: **A)** Illumination geometry and B) calculation of approximate fiber distance for estimating radiant exposure – where d_{fiber} is the optical fiber diameter, r_{fiber} is the optical fiber radius, θ_A is the fiber approach angle, d_{cs+} is the fiber edge's distance off of the surface of the cover slip, and l is the normal distance from the optical fiber face to the cover slip plane.

$$DC = \frac{t_{pulse}}{T_{pulse}}$$

$$P_{peak} = \frac{P_{avg}}{DC}$$

$$E_{pulse} = t_{pulse} \cdot P_{peak}$$
$$E_T = N \cdot E_{pulse}$$
$$E_s = E_T \cdot e^{-\alpha \cdot d_s}$$

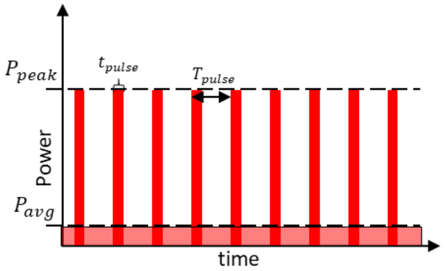

$$D_{spot} = D_{fiber} \cdot 2\,\delta D$$
$$= D_{fiber} + 2(d_s \cdot \tan(\theta))$$

$$RE_{fiber} = \frac{E_T}{\pi \cdot \left(\frac{D_{fiber}}{2}\right)^2}$$

$$RE_{sample} = \frac{E_S}{\pi \cdot \left(\frac{D_{spot}}{2}\right)^2}$$
$$= \frac{E_T \cdot e^{-\alpha \cdot d_s}}{\pi \cdot \left(\frac{D_{fiber} + 2(d_s \cdot \tan(\theta))}{2}\right)^2}$$

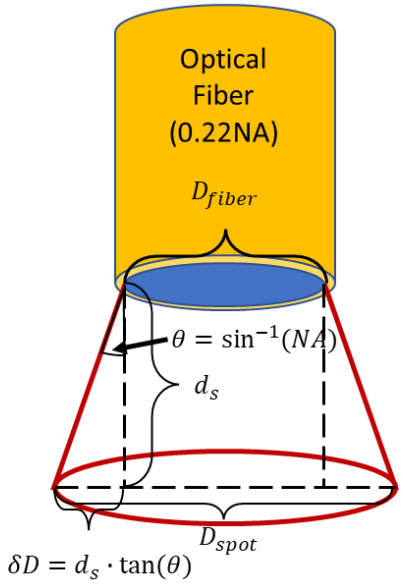

Variable	Definition	Variable	Definition
T_{pulse}	Pulse period, time between pulses	N	Number of pulses delivered
t_{pulse}	Pulse width / duration	α	Absorption coefficient of 1875nm light in water (~26cm^{-1} per Hale and Querry, 1973)
DC	Duty Cycle of IR pulses (0.05)	D_{fiber}	Optical fiber output diameter
P_{peak}	Peak Power of IR pulses	D_{spot}	Effective spot size or diameter (without absorption)
P_{avg}	Average power measured from a train of IR pulses	δD	Change in diameter between sample and fiber face, based on the NA of optical fiber output
E_{pulse}	Optical Energy per IR pulse	θ	Angle of divergence of light from the optical fiber, calculated from the NA of the optical fiber.
E_T	Total Optical Energy	RE_{fiber}	Radiant exposure calculated ex fiber
E_s	Optical Energy observed at the sample, d_s away from fiber output.	RE_{sample}	Radiant Exposure calculated at the sample positioned d_s away from fiber face.
d_s	Distance between fiber output and sample		

Figure S2: Optical dosage calculations at the cell imaging plane based on an absorption-dominated photon distribution in homogenous medium, assuming negligible scattering and non-angled fiber approach to the sample

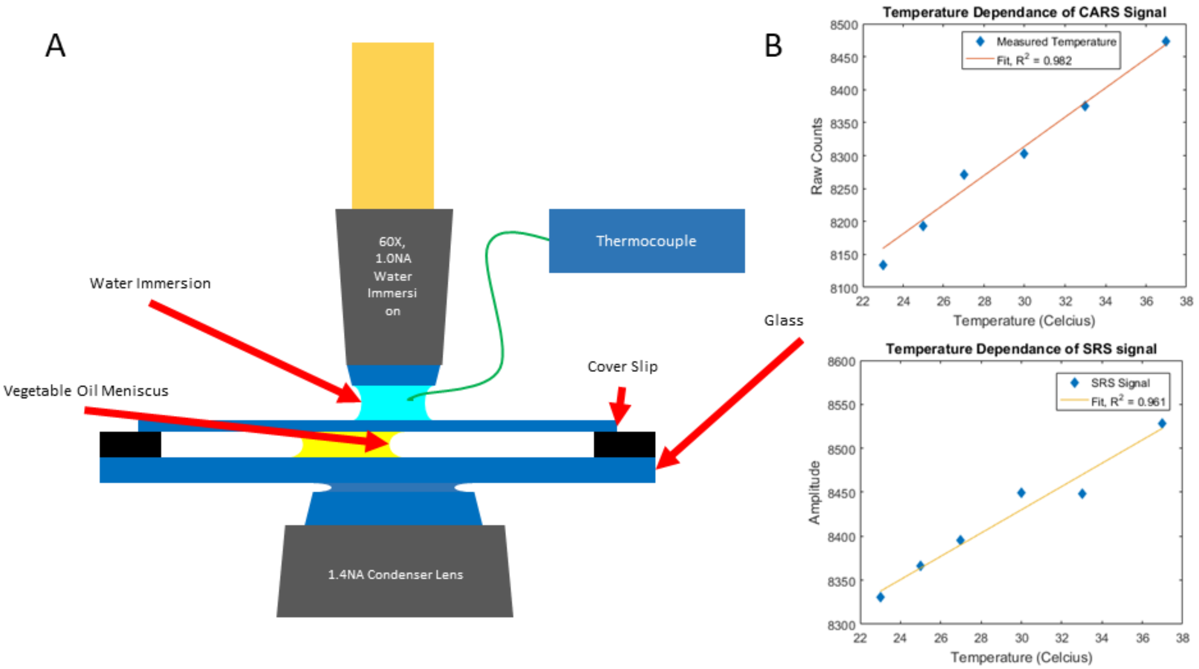

Figure S3: Temperature dependence of 2930 cm^{-1} CARS and SRS signal. A) experimental imaging and temperature measurement setup. B) Raw intensity measurements of vegetable oil meniscus as a function of temperature.

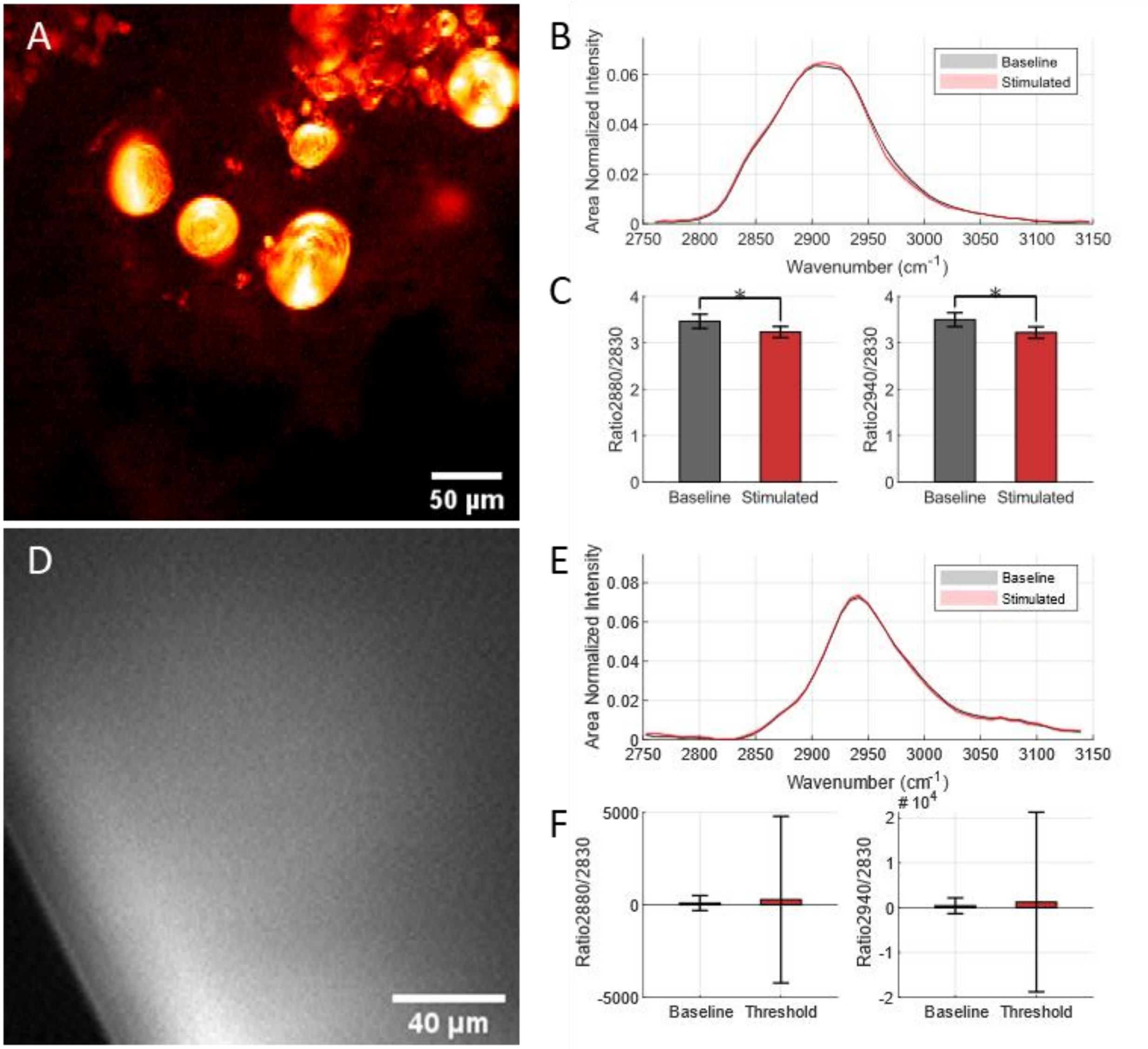

Figure S4: Validation of IR stimulated hsSRS images on isolated control sample preparations of major biological Raman scatterers. (A) SRS image of multi-lamellar vesicles at 2930 cm^{-1} resonance. (B) SRS spectra of baseline and IR-stimulated MLVs. (C, F) Ratiometric comparison of MLVs and BSA SRS spectra, respectively, of resonances indicative of lipid membrane biophysical dynamics. **(**D) SRS image of a 10% bovine serum albumin (BSA) sample in phosphate buffered saline as a control sample to measure protein SRS spectra (E) baseline and IR-stimulated SRS spectra observed in BSA solution.

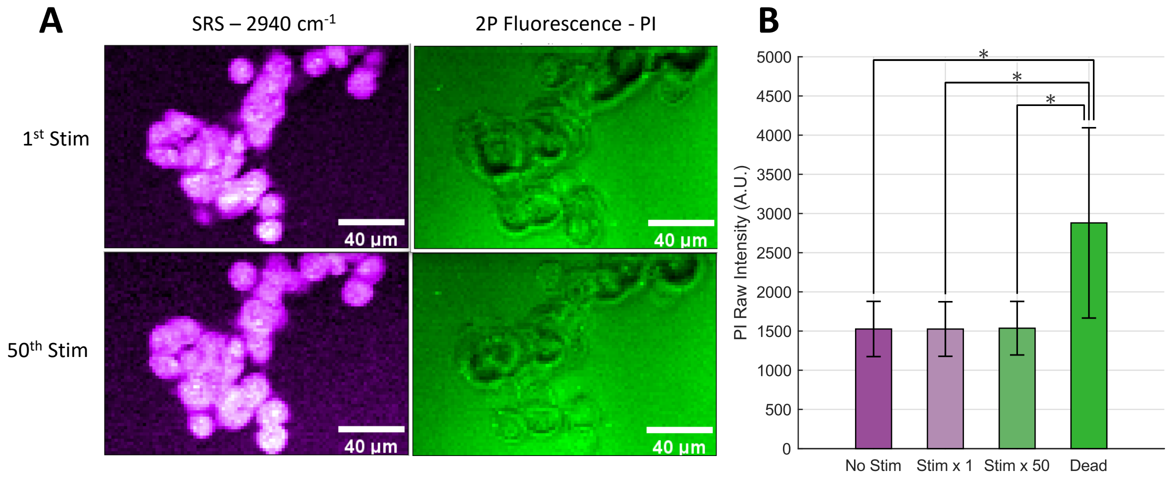

Figure S5: NG108 Cell Viability following hsSRS and repeated INS – (A) Representative average intensity projection images of NG108 cells with SRS (left, magenta) and 2P fluorescence (green, right, identical intensity image scaling) of a cell viability indicator, propidium iodide (PI). Slight differences in cell morphology appear after 50 rounds of INS (bottom) compared to 1 round of INS (top). No substantial update of PI was observable. Scale bars are all 40 µm in width. (B) Intensity level comparison of PI fluorescence in cells exposed to different amounts of threshold INS events. No significant differences observed between non-stimulated and stimulated conditions. Significantly lower fluorescence compared to positive control of dead cells across all conditions. Asterisk indicates $p < 0.05$ based on a 2-sided student's t-test comparisons of cell intensity means and standard deviations across all measured cells ($n = 38$).

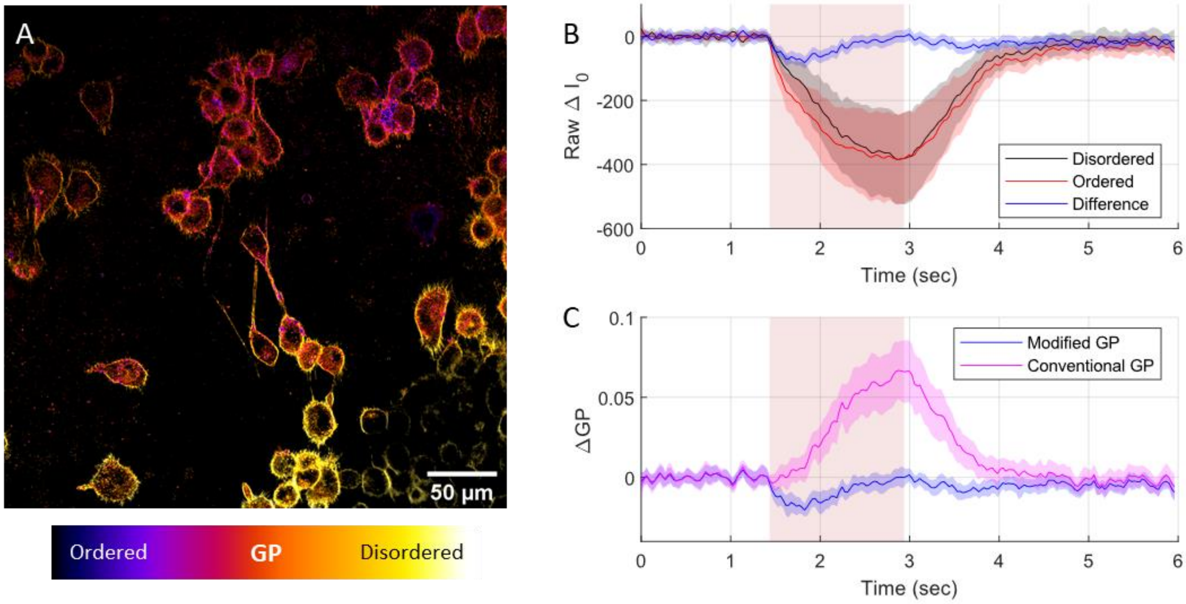

$$GP_{conv}(t) = \frac{(O(t) - D(t))}{(O(t) + D(t))} \quad GP_{mod}(t) = \frac{[O_0 - D_0] + [O(t) - D(t)]}{[O_0 + D_0]}$$

$$O_0 = Ordered\ (Green)\ Channel\ Fluorescence$$

$$D_0 = Disordered\ (Red)\ Channel\ Fluorescence$$

Figure S6: An intensity-invariant metric of general polarization for di-4-ANNEPS imaging of cells during IR stimulation, where signal loss from thermal lensing significantly impacts perceived signal interpretation. A) di-4-ANNEPS loaded NG108 cells. B) Baseline-offset mean detected intensities of mean disordered (black line) and ordered (red line) of all cells in a given experiment, plotted alongside the difference of detected intensities (Ordered – Disordered) C) Calculated conventional general polarization timeseries during IR stimulation alongside adapted general polarization calculation. D) Conventional and adapted GP metric calculations alongside each other. Eliminating the time dependance of the denominator term circumvents the defocusing artifact's impact on the GP calculation.

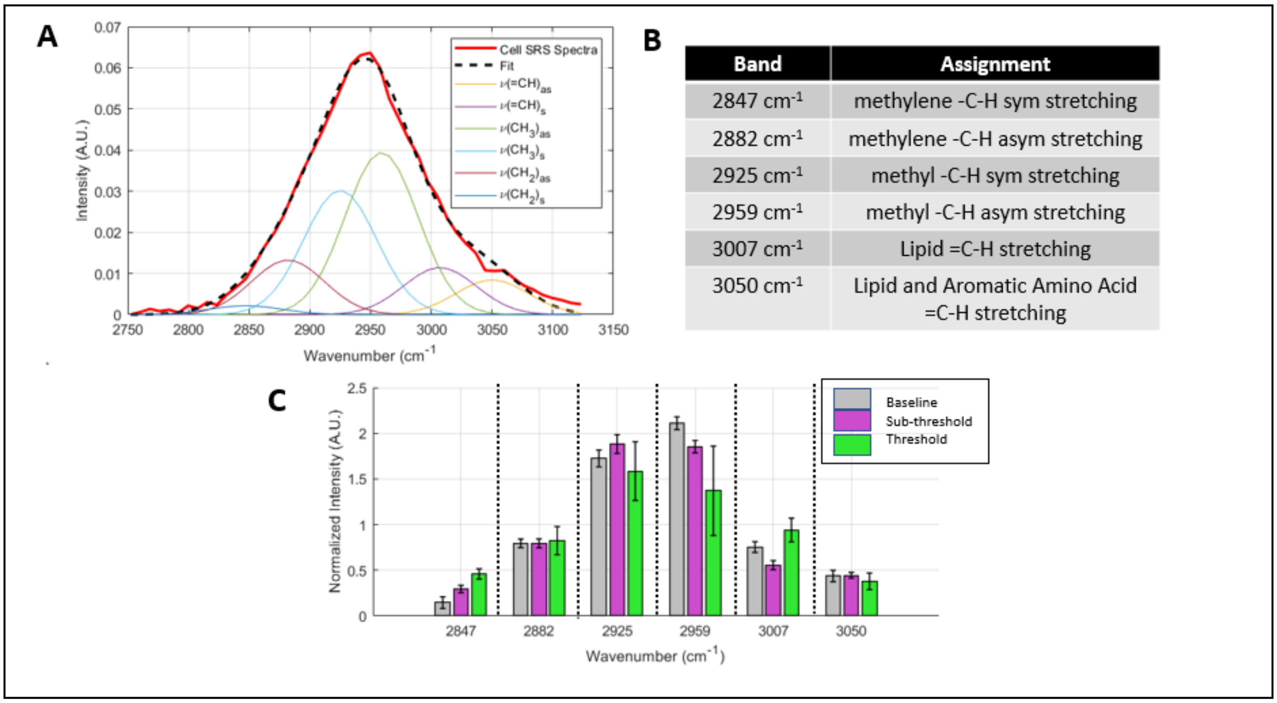

Figure S7: Sub-band analysis of cell SRS spectra based on multi-gaussian curve fitting – A) An example cell spectrum (red) with its corresponding fit (black, dashed) and sub-band constituents. B) Vibrational resonance center frequencies included in the fitting process – derived from Czamara et al. J Raman Spectroscopy 2014. C) Intensity comparison of each sub-band to cell SRS spectra under different stimulation conditions. Error bars represent standard deviation of individual cell spectra.
